# Impact of the Multiple Sclerosis-Associated Genetic Variant *CD226* Gly307Ser on Human CD8 T-Cell Functions

**DOI:** 10.1212/NXI.0000000000200306

**Published:** 2024-09-04

**Authors:** Elena Morandi, Véronique Adoue, Isabelle Bernard, Ekaterina Friebel, Nicolas Nunez, Yann Aubert, Frederick Masson, Anne S. Dejean, Burkhard Becher, Anne Astier, Ludovic Martinet, Abdelhadi Saoudi

**Affiliations:** From the Infinity-Toulouse Institute for Infectious and Inflammatory Diseases (E.M., V.A., I.B., Y.A., F.M., A.S.D., A.A., A.S.), Institut National de la Santé et de la Recherche Médicale (INSERM) UMR 1291, Centre National de la Recherche Scientifique (CNRS) UMR 5051, Université Paul Sabatier (UPS), Toulouse, France; Institute of Experimental Immunology (E.F., N.N., B.B.), University of Zurich, Switzerland; and Cancer Research Center of Toulouse (CRCT) (L.M.), Institut National de la Santé et de la Recherche Médicale (INSERM) UMR 1037, Centre National de la Recherche Scientifique (CNRS), Université Paul Sabatier (UPS), Toulouse, France.

## Abstract

**Background and Objectives:**

The rs763361 nonsynonymous variant in the *CD226* gene, which results in a glycine-to-serine substitution at position 307 of the CD226 protein, has been implicated as a risk factor of various immune-mediated diseases, including multiple sclerosis (MS). Compelling evidence suggests that this allele may play a significant role in predisposing individuals to MS by decreasing the immune-regulatory capacity of Treg cells and increasing the proinflammatory potential of effector CD4 T cells. However, the impact of this CD226 gene variant on CD8 T-cell functions, a population that also plays a key role in MS, remains to be determined.

**Methods:**

To study whether the CD226 risk variant affects human CD8 T-cell functions, we used CD8 T cells isolated from peripheral blood mononuclear cell of 16 age-matched healthy donors homozygous for either the protective or the risk allele of CD226. We characterized these CD8 T cells on T-cell receptor (TCR) stimulation using high-parametric flow cytometry and bulk RNAseq and through characterization of canonical signaling pathways and cytokine production.

**Results:**

On TCR engagement, the phenotype of ex vivo CD8 T cells bearing the protective (CD226-307Gly) or the risk (CD226-307Ser) allele of CD226 was largely overlapping. However, the transcriptomic signature of CD8 T cells from the donors carrying the risk allele presented an enrichment in TCR, JAK/STAT, and IFNγ signaling. We next found that the CD226-307Ser risk allele leads to a selective increase in the phosphorylation of the mitogen-activated protein kinases extracellular signal–regulated kinases 1 and 2 (ERK1/2) associated with enhanced phosphorylation of STAT4 and increased production of IFNγ.

**Discussion:**

Our data suggest that the CD226-307Ser risk variant imposes immune dysregulation by increasing the pathways related to IFNγ signaling in CD8 T cells, thereby contributing to the risk of developing chronic inflammation.

## Introduction

CD226, also known as DNAM-1 (DNAX accessory molecule-1), is a glycoprotein belonging to the Ig superfamily that is constitutively expressed on most of NK cells^1^ and T cells.^1,2^ CD226 is an activating receptor that shares its ligands, CD155 (poliovirus receptor) and CD112 (nectin-2), with 2 negative regulators of lymphocyte signaling, CD96 and TIGIT.^3^ CD226 is involved in the activation and differentiation of T cells.^1,2,4^ In conventional CD4 T cells, CD226 expression is prominent in Th1 and Th17 cells, whereas it is notably decreased in Th2 cells. Blocking CD226 has been shown to improve the progression of experimental autoimmune encephalomyelitis (EAE), an established animal model of multiple sclerosis (MS).^5,6^ By contrast, the role of CD226 is more controversial in CD4 regulatory T-cell functions.^7,8^ In CD8 T cells, CD226 is fundamental for their activation and cytotoxicity, and CD226-negative CD8 T cells exhibit a reduced antitumor response and are dysfunctional when they are activated on T-cell receptor (TCR) engagement.^9^ In accordance with its major role in T-cell functions, CD226 is involved in various pathologic processes such as autoimmune diseases, cancers, and viral infectious diseases.^10,11^

Several studies have associated the genetic variant of CD226 (rs763361T; 307Ser) with increased risk of developing inflammatory diseases including MS.^12^ This disease-associated single-nucleotide polymorphism (SNP) is located in the coding region and generates a glycine-to-serine variation at residue 307 in the cytoplasmic region of CD226. Phenotypically, healthy individuals with the predisposing CD226 genetic variant exhibit decreased surface and gene expression of CD226 in NK cells, as well as effector and regulatory CD4 T cells, compared with carriers of the protective variant.^13^ Functionally, regulatory T cells from healthy individuals carrying the protective genetic variant demonstrated enhanced suppressive capacity.^8^ We previously showed that effector CD4 T cells from healthy individuals homozygous to the risk allele of CD226 produced more IL-17A compared with those bearing the protective allele.^14^ Furthermore, a recent study showed that stably transfected human CD4 T with the risk variant of CD226 exhibited augmented phosphorylation of Tyr322 of CD226 and produced higher levels of IFNγ and TNF.^15^ Moreover, the transfection of mouse myelin Ag-specific CD4 T cells with a mouse/human chimeric CD226 harboring the intracellular region (residues 286–336) of the human risk variant exacerbated EAE pathogenicity.^15^ These findings indicate that the CD226 risk allele could potentially play a significant role in predisposing individuals to CNS autoimmunity through mechanisms involving the reduction of immune-regulatory capacity in Tregs and the elevation of proinflammatory potential in effector CD4 T cells. There is increasing acknowledgment of the significant involvement of CD8 T cells in MS. For example, histopathologic examinations of postmortem brain tissue reveal that CD8 T cells outnumber CD4 T cells in MS lesions.^16,17^ Moreover, CD8 T-cell lines reactive to myelin have been demonstrated to induce EAE.^18,19^ Nevertheless, the effect of the CD226 risk allele on CD8 T-cell functions remains to be elucidated.

In this study, we combined phenotypic and functional studies to characterize the impact of the risk allele of CD226 on human CD8 T-cell functions. We showed that CD8 T cells from healthy donors carrying the risk variant of CD226 exhibit an increase in the pathways related to IFNγ signaling. This is likely contributing to the enhanced inflammation observed in immune-mediated diseases with which the risk allele of CD226 has been associated.

## Methods

### Human PBMC

Peripheral blood mononuclear cells (PBMCs) from healthy blood donors were prepared by gradient centrifugation (MLS-Ficoll, Eurobio, Les Ulis, France) of buffy coats and kept frozen in liquid nitrogen. We conducted genotyping for the CD226 SNP rs763361 using the TaqMan SNP Genotyping Assay from Applied Biosystems following the manufacturer's instructions. Individuals carrying the rs763361T allele, which results in CD226-307Ser expression, were classified as being at risk, whereas those carrying the rs763361C allele, leading to CD226-307Gly expression, were considered to have the protective allele. Experiments were performed on cells from male donors who were homozygous for either allele.

### CD8 T-Cell Isolation and Activation

Frozen PBMCs were thawed and left ON at 37°C, and monocytes were removed by plastic adherence. CD8 T cells were purified by negative selection using the EasySep Human CD8+ T Cell Isolation Kit (Stemcell) or sorted by flow cytometry. The percentage of residual CD4 T cells after depletion was always less than 0.5%, and the remaining population consisted of 95–98% of CD8 T cells. Isolated CD8 T cells were cultured in complete medium RPMI (10% fetal calf serum, 1% hepes, 1% sodium pyruvate, 1% nonessential amino acids, 1% penicillin-streptomycin, 1% l-glutamine) for 3 days in the presence of 1 μg/mL coated anti-CD3 mAb (clone TR66, produced in house) with or without 1 μg/mL soluble anti-CD28 mAb (clone CD28.2, BD) or IL-12 (Prepotech). After 3 days of activation, cell culture supernatants were frozen at −20°C. Cytokine secretion was assessed using the CD8/NK panel LEGENDplex Immunoassay (Biolegend) following the manufacturer's instructions.

### Flow Cytometry

Surface staining was performed for 30 minutes at 4°C in the dark. Cells were then fixed and permeabilized using the Fix/Perm kit (BD) for 30 minutes at RT and were stained for intracellular markers for 30 minutes at RT. For cytokine production, the cells were stimulated for 4 hours with 100 ng/mL of PMA, 1 μg/mL of ionomycin, and 1 μg/mL of the GolgiPlug monensin (BD Biosciences). Between each step, cells were washed twice with fluorescence-activated cell sorting buffer containing 2% FCS. The live/dead marker (ThermoFisher Scientific) was included in the staining to assess the viability. Proliferation was measured using the Cell Trace Violet kit (ThermoFisher) following the manufacturer's instructions. Isotype controls and fluorescence minus one samples were used as control and to set the gating. Cells were analyzed by flow cytometry using the Fortessa and LSRII flow cytometers (BD Biosciences, USA) and FlowJo software (version V10, FlowJo, LLC, USA).

### RNA Sequencing

Fluorescence-activated cell-sorted CD3^+^CD8^+^ cells were cultured in complete medium RPMI for 0, 4, and 24 hours in the presence of 1 μg/mL coated anti-CD3 mAb (clone TR66, produced in-house) and 1 μg/mL soluble anti-CD28 mAb (clone CD28.2, BD). RNA was extracted using the RNeasy Micro Kit (Qiagen) following the manufacturer's instructions. Libraries were prepared using the Illumina Stranded Total RNA Prep kit starting from 7.5 ng of total RNA and using 14 PCR cycles for library amplification. The kit for the index sequences used was IDT Illumina RNA UDI A Lig 96 Indexes 96 Spl. Library pool quantification and sequencing were performed at the GeT-Santé (I2MC, Toulouse, France) and GeT-PlaGe (INRAE, Toulouse, France) core facilities, respectively. The pool was quantified by qPCR using the KAPA Library Quantification Kit (Roche, Basel, Switzerland) to obtain an accurate quantification. Subsequently, sequencing was conducted on a single S4 lane of the Illumina NovaSeq 6000 instrument (Illumina, San Diego, USA), using the NovaSeq 6000 S4 v1.5 Reagent Kit (300 cycles) and a paired-end 2 × 150 pb strategy. Each library yielded between 20 and 44 million paired-end raw reads.

### Simple Western

Primary isolated CD8 cells were expanded on activation with phytohemagglutinin (PHA) in complete medium RPMI for 10 days in the presence of 100U/mL IL-2 (Prepotech). Expanded CD8 T cells were then stimulated with either 0.5 μg/mL anti-CD3 (UCHT-1, BD) and 1 μg/mL anti-CD28 (clone CD28.2, BD) monoclonal antibodies or LEAF-purified mouse IgG1 isotype control. Cells were incubated with the specified antibodies for 30 minutes on ice, followed by washing with an ice-cold medium and suspension in a warm medium for 5 minutes at 37°C. Antibodies were cross-linked using the goat anti-mouse Fab2 secondary antibody (20 μg/mL; Jackson ImmunoResearch) for the indicated time. Stimulation was stopped by adding 2x lysis buffer containing 2% Triton X-100 and phosphatase inhibitors. Protein phosphorylation was assessed by Jess Replex Simple Western with phosphospecific antibodies for ERK1/2 phosphorylated at Thr202/Tyr204 (D123.14.4E), for serine/threonine protein kinase (AKT) phosphorylated at Ser473 (D9E), and for P38 phosphorylated at Thr180/Tyr182 (rabbit polyclonal antibody). The total amount of proteins was measured using the antibodies anti-ERK (3A7), anti-AKT (GSK-3B), and anti-P38 (rabbit polyclonal antibody). All antibodies were from Cell Signaling Technology. Signals were analyzed using Compass software.

### Phosflow STAT

Activated CD8 T cells with anti-CD3 and anti-CD28 (1 μg/mL) Abs for 3 days were restimulated in the presence of IL-12 (50 ng/mL, R&D), IFNγ (50 ng/mL, Prepotech), and IFNα (10,000 U/mL, R&D) for 15 minutes at 37°. The reaction was stopped by adding the cold fixation buffer (eBioscience) and incubating for 50 minutes. Cells were permeabilized using the Phosflow Perm Buffer III (BD) for 15 minutes. The staining was performed with anti-Stat4 pY693 (clone 38/P, BD) and anti-Stat1 pY701 (clone 4a, BD). Cells were analyzed by flow cytometry using Fortessa (BD Biosciences) and FlowJo software (version V10, FlowJo, LLC).

### Data Analysis

Data are presented as means ± SEM. The GraphPad Prism statistical package was used for statistical analyses (GraphPad Software, Inc). Results were compared by the Mann–Whitney test or 2-way ANOVA with Bonferroni post-test analysis where more conditions were tested. *P* values are indicated in the figures, and results were considered statistically significant when *p* < 0.05.

### Standard Protocol Approvals, Registrations, and Patient Consents

PBMCs were isolated from buffy coat preparations sourced from willing healthy blood donors at the blood bank of Purpan University Hospital in Toulouse, France. The research received approval from the Ethical Committee for the French South-West and Overseas region and was registered with the French Ministry of Higher Education and Research under reference number DC-2015-2488.

### Data Availability

Information omitted from the article due to space constraints can be shared (with anonymization) on request from any qualified researcher for the purpose of replicating procedures and results. Newly generated sequencing data have been deposited on GEO under the accession number GEO GSE266132.

## Results

### Risk Variant of CD226 Does Not Alter the Phenotype of CD8 T Cells

**Figure 1:**
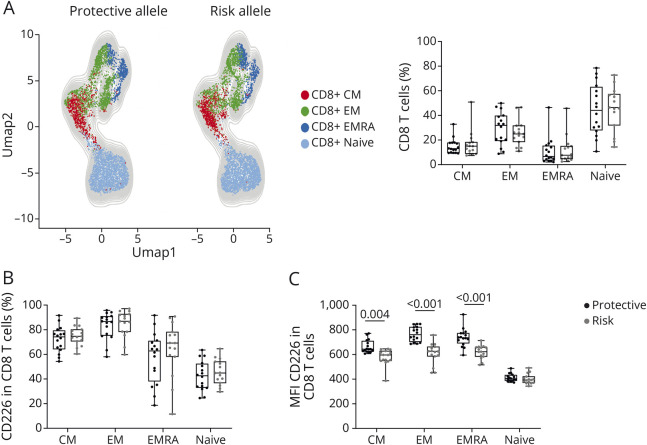
Phenotype of Ex Vivo CD8 T Cells Carrying the Protective or the Risk Variant of CD226 PBMCs from healthy donors homozygous for either the protective (CD226-307Gly, N = 16) or the risk (CD226-307Ser, N = 16) allele were analyzed ex vivo by flow cytometry. (A) Uniform Manifold Approximation and Projection (UMAP) and frequency of central memory (CM, CD45RA- CCR7+), effector memory (EM, CD45RA- CCR7-), memory re-expressing CD45RA (EMRA, CD45RA+ CCR7-), and naïve (CD45RA+ CCR7+) CD8 T cells are shown for each allele. (B) Frequency and (C) MFI of CD226 expression are plotted in CM, EM, EMRA, and naïve CD8 T cells. MFI = mean fluorescent intensity; PBMCs = peripheral blood mononuclear cells.

The specific function of a single polymorphism in patients is challenging because of the influence of other risk genes and therapies. This complexity is highlighted by a study on the CD226 allele, which showed differences between risk and protective alleles in Tregs from healthy controls while Tregs from patients with MS behaved like those from healthy controls with the risk allele, suggesting that protective haplotype effects are nullified in the context of ongoing autoimmune disease.^8^ It is for this reason that we decided to analyze the impact of the CD226Gly307Ser variant on CD8 T-cell functions in selected PBMCs from 16 donors homozygous for either allele. We compared the phenotype of CD8 T cells carrying the risk (CD226-307Ser) or the protective (CD226-307Gly) allele ex vivo by flow cytometry. We analyzed the distribution of the different CD8 T subsets, their expression of T-cell related markers and transcription factors, and their ex vivo production of cytokines after stimulation with PMA/ionomycin. We did not find any significant differences concerning the frequency of naïve, central memory (CM), effector memory (EM), or effector re-expressing CD45RA (EMRA) CD8 T cells (Figure 1A) between both genotypes. The frequency of CD226+ cells was comparable across both genotypes within the 4 CD8 T subsets (Figure 1B). TEMRA, EM, and CM CD8 T cells carrying the risk allele (CD226-307Ser) expressed a reduced level of CD226 in terms of mean fluorescent intensity (MFI) when compared with the protective allele (CD226-307Gly) (Figure 1C), similar to what was previously reported for CD4 T cells.^4^ Unsupervised clustering (eFigure 1, A and B) based on the expression of T cell–related markers (CD25, CD69, HLA-DR, CD27, TCR, CD28, KLRG1, ICOS, CD57, PD-1, 2B4, CD11b, OX-40, Ki67, CTLA-4) and transcription factors (FOXP3, EOMES, T-BET) revealed only 2 clusters being significantly different between the 2 genotypes. Cluster 13 (1.33% of CD8 T cells), characterized by a high expression of CD45RA and CD226 and medium expression of CD27, TIGIT, and EOMES, was increased in CD8 T cells carrying the CD226-307Ser variant (eFigure 1C). On the contrary, cluster 20 (0.28% of CD8 T cells), characterized by a high expression of CD226, CD27, and PD1 and medium expression of TIGIT, was decreased in CD8 T cells carrying the CD226-307Ser variant (eFigure 1D). The differential presence of these clusters could represent a different immune regulation influenced by the CD226 allele, but their very low frequency (<1.5%) suggests that the CD226-307Ser variant has not a major impact on the phenotype of CD8 T cells. We, therefore, decided to investigate the impact of the CD226-307Ser variant on the transcriptome of the ex vivo CD8 T cells after activation.

### Risk Variant of CD226 Alters the Transcriptional Signature of CD8 T Cells

**Figure 2:**
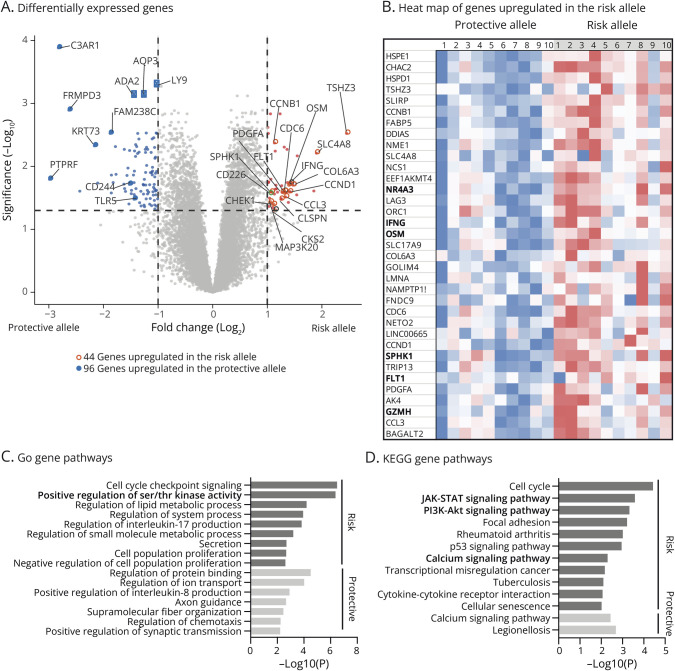
Transcriptomics of CD8 T Cells Carrying the Protective or the Risk Variant of CD226 RNA sequencing of purified CD8 T cells from healthy donors homozygous for either the protective (CD226-307Gly, N = 10) or risk (CD226-307Ser, N = 10) allele stimulated with anti-CD3 and anti-CD28 mAbs for 0 and 24 hours. (A) Volcano plot representation of differential gene expression analysis after 24 hours of activation. Colored points show the differentially expressed genes (DEGs) (fold change >2 and Padj <0.05). In red are depicted the genes upregulated in the risk allele and in blue the genes upregulated in the protective allele. (B) The heat map shows the relative expression of the most upregulated genes in the risk allele for each sample. Metascape analysis based on DEGs and showing the pathways upregulated in the risk or protective allele using the gene ontology (C) and the Kyoto Encyclopedia of Genes and Genomes (D) pathway databases.

Signals transmitted by the TCR are regulated by coreceptors, which selectively influence distinct signaling pathways and contribute to the differentiation and effector characteristics of T cells. It is important to note that it was previously shown that CD3/CD28 costimulation activates CD226 independently of CD226 ligation.^2,4,20,21^ In addition, CD8 T cells that do not express CD226 are anergic and do not respond to the TCR engagement.^9^ Thus, we assessed the transcriptomic changes associated with the CD226Gly307Ser variant using CD8 T cells purified from 10 donors homozygous for either allele that were either unstimulated (“0 hours”) or stimulated with anti-CD3 and anti-CD28 mAbs for 4 hours or 24 hours. We then performed bulk RNA sequencing (RNAseq) and selected the statistically differentially expressed genes (DEGs, Padj <0.05 and fold change >2) between CD8 T cells carrying the protective or the risk variant of CD226 at all time points. We did not detect any significant DEGs in the ex vivo unstimulated T cells and on 4 hours of activation. By contrast, we detected 44 upregulated genes in the risk allele and 96 in the protective allele after 24 hours of activation (Figure 2A). Of interest, among the differentially overexpressed genes with the risk allele (Figure 2, A and B), we found IFNγ and Granzyme H, 2 key molecules for CD8 T-cell functions. Of note, the nuclear orphan receptor subfamily 4 group A member 3 (NR4A3), triggered by TCR signaling and related to CD8 T-cell differentiation and effector function,^22^ was also upregulated with the risk allele. Other interesting upregulated genes related to T-cell signaling were oncostatin M (OSM), sphingosine kinase 1 (SPHK1), and Fms-related receptor tyrosine kinase 1 (FLT1). OSM encodes a cytokine and growth regulator that is part of the cytokine production signaling regulated by ERK and AKT^23^ while SPHK1 catalyzes the phosphorylation of sphingosine to form sphingosine 1-phosphate that promotes the activation of NF-kappa-B and p38 mitogen-activated protein kinase (MAPK) signaling.^24^ FLT1 encodes a tyrosine-protein kinase that is a member of the vascular endothelial growth factor receptor family, leading to the activation of phospholipase C gamma, MAPK, and AKT signaling pathways.^25^

To better understand the overall impact of the CD226-Gly307Ser variant on CD8 T-cell transcriptome after 24 hours of stimulation, we performed gene ontology (Figure 2C) and Kyoto Encyclopedia of Genes and Genomes enrichment analyses (Figure 2D). Results revealed increased expression of genes involved in regulation of protein kinase activity, JAK-STAT and PI3K-AKT signaling, calcium signaling pathway, cytokine-cytokine receptor interaction, and cellular senescence. To explore whether certain signaling pathways began to be deregulated after 4 hours of stimulation, we performed the gene set enrichment analysis (GSEA) that uses a different and complementary methodology to differential expression analysis (eFigure 2). Of note, it confirmed the enrichment of genes involved in T-cell receptor (eFigure 2A) and JAK-STAT signaling pathways (eFigure 2B) already detected at 24 hours. Moreover, we also found an enrichment of genes involved in “positive regulation of IFNγ production” in the CD8 T cells from donors carrying the risk allele compared with those carrying the protective allele (eFigure 2C). These results suggest that on CD3 and CD28 stimulation, CD8 T cells carrying the risk allele are transcriptionally more prone to support TCR signaling, JAK-STAT pathways, and IFNγ production.

### Risk Allele of CD226 Selectively Increases the Phosphorylation of ERK1/2 in CD8 T Cells

**Figure 3:**
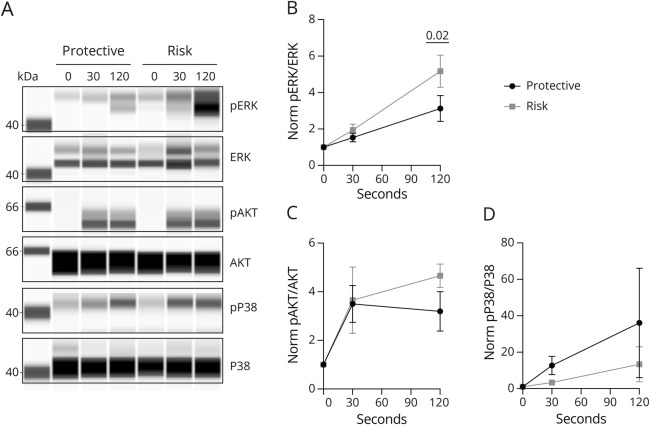
TCR Signaling of CD8 T Cells Carrying the Protective or the Risk Variant of CD226 Expanded CD8 T cells from healthy people homozygous for either the protective (CD226-307Gly, N = 6) or risk allele (CD226-307Ser, N = 6) were stimulated with anti-CD3 and anti-CD28 mAbs for 0, 30, and 120 seconds. Phosphorylation of ERK, AKT, and p38 was detected through Jess Replex Simple Western (A) and quantified. The ratio between the phosphorylated and total protein is shown in the graphs (B, C, and D). 2-way ANOVA was used for statistical analysis.

In murine thymocytes, the deletion of CD226 affects the TCR-induced activation of AKT, ERK1/2 (MAPK3/1), NF-KB, and P38 (MAPK14) signaling^26^ suggesting that CD226 fine-tunes TCR signaling. Thus, we compared the TCR signaling of expanded human CD8 T cells carrying the CD226 protective or risk variant after the engagement of the TCR. We showed that the CD226 risk allele led to a selective increase in the phosphorylation of the MAPKs extracellular signal–regulated kinases 1 and 2 (ERK1/2) after TCR stimulation of CD8 T cells (Figure 3, A and B). In comparison, the CD226 risk allele did not affect significantly the extent of AKT (Figure 3, A and C) and MAPK p38 (Figure 3, A and D) phosphorylation indicative of its specific effect on TCR signaling pathways. Because ERK is one of the main signaling molecules involved in different T-cell programs, such as activation, proliferation, differentiation, and cytokine production, we next sought to investigate whether the impact of the Gly307Ser variant of CD226 on ERK activation could be associated with changes in CD8 T-cell functions.

### CD226 Risk Allele Confers Elevated IFNγ Production by CD8 T Cells

To analyze the impact of the CD226 Gly307Ser variant on CD8 T-cell functions, we isolated CD8 T cells and stimulated them for 3 days with plate-bound anti-CD3 mAb alone or in combination with anti-CD28 mAb. The cells were then analyzed for proliferation, viability, activation markers, and transcription factor expression by flow cytometry. In addition, the cytokine production in culture supernatants collected after 3 days of stimulation was determined by CBA.

**Figure 4:**
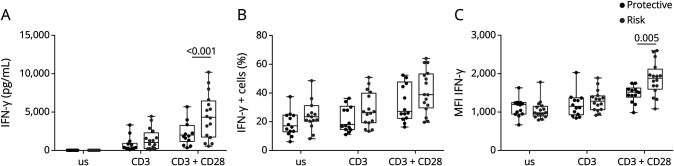
Functions of CD8 T Cells Carrying the Protective or the Risk Variant of CD226 Functional analysis of CD8 T cells isolated from PBMCs of healthy donors carrying either the protective (CD226-307Gly, N = 16) or risk (CD226-307Ser, N = 16) allele stimulated with anti-CD3 mAb alone or in combination with anti-CD28 mAb for 3 days. (A) IFNγ levels in culture supernatants were measured using LEGENDplex. Percentage (B) and MFI (C) of IFNγ+ CD8 T cells detected by flow cytometry after stimulation with PMA and ionomycin. 2-way ANOVA was used for statistical analysis. MFI = mean fluorescent intensity; PBMCs = peripheral blood mononuclear cells.

We did not find any significant differences in CD8 T-cell viability and proliferation between the 2 genotypes (eFigure 3A). The frequency of CD226-positive CD8 T cells increased on activation with a higher trend in the CD8 T cells from the risk group while the MFI was similarly augmented (eFigure 3B). CD8 T cells from both groups expressed similar frequencies and MFI of the activation markers CD69 and PD1 indicative of comparable activation levels (eFigure 3, C and D). In addition, the expression of the receptor TIGIT was not different between the 2 groups (eFigure 3E). Among all cytokines tested, only IFNγ was significantly increased in the supernatant of CD8 T cells expressing the risk allele (Figure 4A). By contrast, the production of TNF, IL-17, IL-10, perforin, GranzB, GranzA, and FasL was not statistically different between the 2 genotypes (eFigure 4). To investigate whether the increased levels of IFNγ in the supernatants were the consequence of an increased secretion at the cell level or an increased frequency of IFNγ producing CD8 T cells, we analyzed the IFNγ expression by flow cytometry using intracytoplasmic staining. Although there was only a tendency for an increased proportion of cells producing IFNγ (Figure 4B), the IFNγ MFI was increased in CD8 T cells expressing the CD226-307Ser variant (Figure 4C), suggesting that individual CD8 T cells carrying the risk allele had a higher ability to produce IFNγ on activation with anti-CD3 and anti-CD28 mAbs. This result is in line with the positive regulation of the IFNγ production pathway found in our RNAseq analysis and confirms the increased capacity of IFNγ secretion by CD8 T cells harboring the risk variant after TCR activation.

### Risk Allele of CD226 Promotes IL-12Rβ2/STAT4/IFNγ Pathways in CD8 T Cells

**Figure 5:**
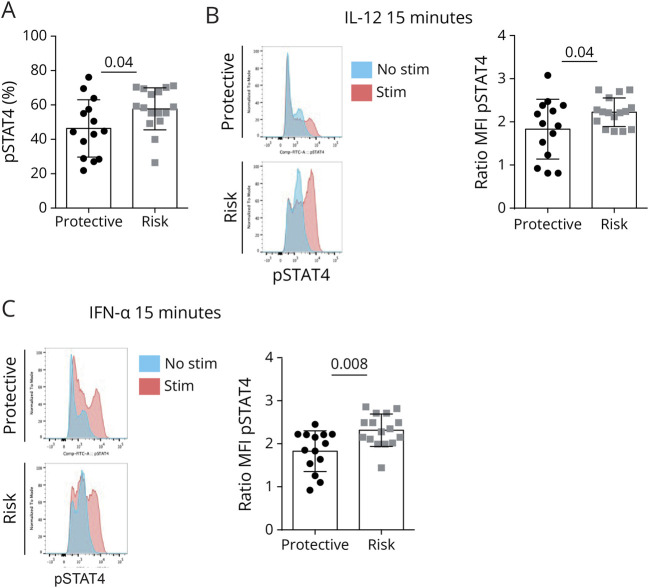
IFNγ Pathway in CD8 T Cells Carrying the Protective or the Risk Variant of CD226 Phosphorylation of STAT4 was measured as % and MFI through Phosflow in CD8 T cells isolated from healthy donors carrying either the protective (CD226-307Gly, N = 16) or risk (CD226-307Ser, N = 16) allele that was stimulated with anti-CD3 and CD28 Abs alone (A) or with addition of IL-12 (B) or IFNα (C) for 15 minutes. The graph bar shows the ratio between IL-12-stimulated and unstimulated cells. The Mann-Whitney test was used for statistical analysis. MFI = mean fluorescent intensity.

The signaling pathways that contribute to IFNγ production have been well characterized. IFNγ receptor (IFNGR) signaling activates the signal transducer and activator of transcription 1 (STAT1), which in turn induces the expression of T-BET in effector T cells.^27^ This leads to the expression of the β2 chain of IL-12 receptor (IL-12Rβ2) that induces the phosphorylation of STAT4 and amplifies the production of IFNγ.^28^ To study the mechanisms by which the CD226 Gly307Ser polymorphism modulates IFNγ production by CD8 T cells, we measured by flow cytometry the expression of IFNγR1 and IL-12R and the phosphorylation of STAT1 and STAT4 in CD8 T cells on activation. We first analyzed whether the effects of CD226 variants on IFNγ secretion were associated with a differential IFNγR-mediated signaling. We showed that the expression of IFNγR and the transcription factor T-BET was similar between activated CD8 T cells of both genotypes (eFigure 5, A and B). The phosphorylation of STAT1 after the activation with anti-CD3 and anti-CD28 Abs (eFigure 5C) and on further incubation for 15 minutes with IFNγ (eFigure 5D) or IFNα (eFigure 5E) was also comparable between both genotypes, suggesting that the CD226 variant operates independently of IFNγR-mediated signaling. The cell surface abundance of the β1 (eFigure 5F) and β2 chains (eFigure 5 G) of the IL-12R was comparable in CD8 T cells carrying the risk or the protective allele of CD226, indicating that the difference in IFNγ secretion was not the consequence of an alteration of IL-12 receptor expression. By contrast, on TCR engagement, the proportion of pSTAT4-positive cells was higher in CD8 T cells carrying the CD226 risk allele compared with CD8 T cells with the protective one (Figure 5A). Restimulating the cells for 15 minutes in the presence of IL-12 further increased the phosphorylation of STAT4 (Figure 5B). We obtained a similar result on stimulation in the presence of IFNα for 15 minutes, suggesting that this observation is not specific to IL-12, rather related to STAT4 itself (Figure 5C).

**Figure 6:**
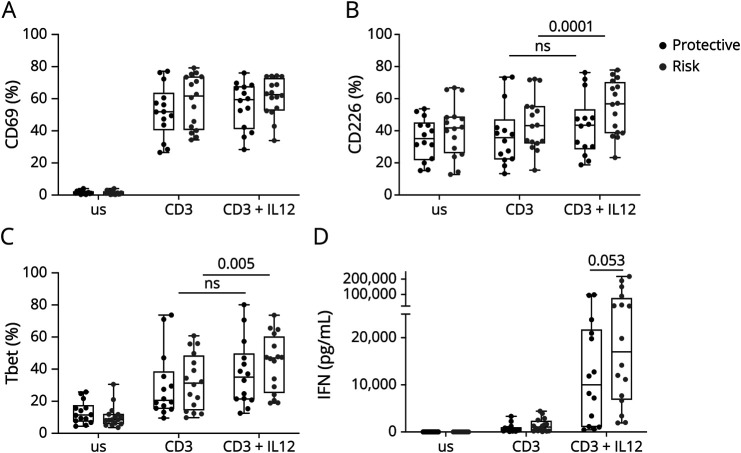
Functionality of CD8 T Cells Carrying the Protective or the Risk Variant of CD226 in Th1-Polarized Condition Isolated CD8 T cells from healthy donors carrying either the protective (CD226-307Gly, N = 16) or risk (CD226-307Ser, N = 16) allele were stimulated with anti-CD3 mAb alone or in combination with IL-12 for 3 days. Frequency of CD69 (A), CD226 (B), and T-BET (C) expression by CD8 T cells was analyzed with flow cytometry. (D) Production of IFNγ was measured in the culture supernatants using the LEGENDplex CBA.

**Figure 7:**
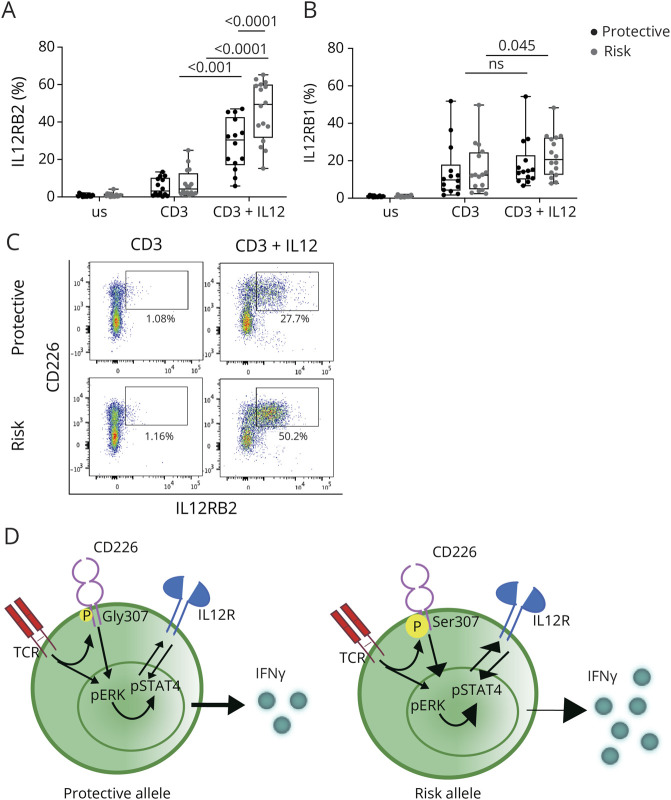
Expression of IL-12R by CD8 T Cells Carrying the Protective or the Risk Variant of CD226 in Th1-Polarized Condition Isolated CD8 T cells from healthy donors carrying either the protective (CD226-307Gly, N = 16) or risk (CD226-307Ser, N = 16) allele were stimulated with anti-CD3 mAb alone or in combination with IL-12. Frequency of IL-12Rβ2 (A) and IL-12Rβ1 (B) expression by CD8 T cells was detected by flow cytometry after 5 days of activation. (C) Dot plot showing the expression of CD226 and IL-12Rβ2 by CD8 T cells harboring the protective or risk allele of CD226 after 5 days of activation. 2-way ANOVA was used for statistical analysis. (D) Proposed model on the consequence of the TCR activation with CD226 carrying the protective (left panel) or risk (right panel) allele in CD8 T cells. The engagement of the TCR induces phosphorylation of CD226 that synergizes into the TCR signaling pathways, leads to phosphorylation of STAT4, and results in IFNγ production. In CD8 T cells from donors carrying the risk variant, these pathways are amplified contributing to the risk of developing chronic inflammation.

We next tested whether these alterations of the IFNγ pathway were still observed on CD8 T-cell activation under Tc1 polarization conditions. Thus, we activated total CD8 T cells with anti-CD3 mAb and IL-12 for 3 or 5 days. The expression of CD69 was similar between the 2 groups indicating comparable activation (Figure 6A, eFigure 6A). Compared with stimulation with CD3 alone, the addition of IL-12 significantly increased the frequency of CD226+ (Figure 6B) and T-BET+ (Figure 6C) only in the CD8 T cells carrying the risk allele. The level of expression of CD226 (eFigure 6B) and T-BET (eFigure 6C) was similar between both genotypes. Secretion of IFNγ was drastically increased on IL-12 supplementation, and this was again higher in the supernatant of CD8 T cells expressing the risk allele (Figure 6D). After addition of IL-12, we found that the IL-12Rβ2 chain, known to be regulated by STAT4, was upregulated with a strong difference between the 2 CD226 alleles especially after 5 days of activation (Figure 7A, eFigure 6D). By contrast, in this experimental setting, the IL-12Rβ1 was slightly sensitive to IL-12 (Figure 7B, eFigure 6E). Of interest, after IL-12 addition, all cells that expressed the IL-12Rβ2 on the addition of IL-12 were CD226+ (Figure 7C), suggesting that CD226 is directly involved in the IL-12Rβ2/STAT4/IFNγ pathway and that this pathway is amplified in donors expressing the CD226 risk variant (Figure 7D).

## Discussion

Mounting appropriate T cell–driven immune responses requires the integration of a multitude of signals that arise from various types of receptors. The TCR signaling pathways induced after antigen stimulation are fine-tuned by costimulatory molecules to adjust the sensitivity of T cells to antigen stimulation in given immunologic contexts. Consequently, the polymorphisms in gene coding for these costimulatory molecules can affect their expression and function, leading to the dysregulation of the immune response. This highlights the importance of performing functional studies using the genetic variants of these costimulatory molecules to understand the mechanisms by which they influence the immune response.

In this study, we investigated the impact of the CD226 Gly307Ser polymorphism on CD8 T cells at the phenotypic, transcriptomic, and functional levels. Ex vivo CD8 T cells from donors carrying the risk variant do not exhibit tangible phenotypical differences compared with cells from donors expressing the protective variant, except for a decreased expression of CD226 in the ex vivo effector/memory CD8 T cells. This observation is in line with what has been already reported in NK, effector, and regulatory CD4 T cells.^13,29^ CD8 T-cell activation is mainly triggered by TCR recognition of MHC-I-peptide complexes, and costimulatory/inhibitory molecules play a fundamental role in fine-tuning CD8 T-cell activation. Indeed, it has been shown that CD8 T cells that do not express CD226 are less responsive to TCR stimulation,^9^ and a recent study showed that deletion of CD226 in murine thymocytes impaired TCR activation signaling and diminished AKT, ERK, NF-κB, and p38 phosphorylation levels,^26^ suggesting that CD226 contributes to these TCR signaling pathways. Converging evidence suggests that CD226 may synergize with TCR signaling in a CD122/CD155-independent manner.^2,4,21^ These ligand-independent mechanisms are not unusual because several transmembrane receptors including chemokine receptors^30,31^ and costimulatory molecules^32,33^ were shown to be transactivated on TCR ligation and to increase T-lymphocyte activation independently of their ligands. For example, TCR ligation was shown to induce transactivation of CXCR4 in the absence of its ligand CXCL12 resulting in the formation of TCR-CXCR4 complex that increases *IL2* mRNA stability and subsequent T-cell proliferation.^30,31^ In line with these studies, we detected in CD8 T cells expressing the risk allele of CD226 an increased expression of transcripts regulating TCR signaling on activation with anti-CD3 and anti-CD28 Abs. We tested the phosphorylation of some canonical signaling molecules and showed that the CD226 risk allele leads to a selective increase in the phosphorylation of ERK after TCR stimulation of CD8 T cells. In comparison, the CD226 risk allele did not affect the extent of phosphorylation of the MAPK p38, indicative of the specific effect of the CD226 genetic variant on TCR signaling pathways. Because ERK2 selectively enhances the production of Th1 cytokines without affecting T-BET abundance,^34^ we speculated that the CD226 risk allele might favor IFNγ production by CD8 T cells through ERK activation. Of interest, related to ERK pathways, in the RNA sequencing data, we also found an enrichment in IFNγ and STAT gene signature. In accordance, we detected an increased phosphorylation of STAT4 by flow cytometry and the increased production of IFNγ in the supernatants of stimulated CD8 T cells expressing the risk variant. Together, these data show that the CD226-307Ser risk variant amplifies the IFNγ pathways in CD8 T cells.

IFNγ is one of the major cytokines produced by CD8 T cells in the context of infection, tumor control, and autoimmunity. The main signaling pathways that induce IFNγ production by T cells are mediated by cytokine activation of the JAK-STAT family. In particular, IFNγ itself, IFNα, and IL-12 have been shown to be implicated in this mechanism. IFNγ binds to the heterodimeric IFNγR that leads to the downstream tyrosine phosphorylation of STAT1 to induce expression of IFNγ-regulated genes and T-BET. IL-12 binds to IL-12R inducing STAT4 phosphorylation leading to its dimerization and translocation to the nucleus, where it binds the DNA and amplifies the expression of IFNγ. IFNα binds to IFNAR, resulting in tyrosine phosphorylation of both STAT1 and STAT4 complexes. In our model, both IL-12 and IFNα induce the increased phosphorylation of STAT4 in the risk allele compared with the protective allele, suggesting an important role of CD226 in the STAT4 pathway independent of the cytokine used to stimulate it. The addition of IL-12 on activation with anti-CD3 mAb led to an amplification of IFNγ production and IL-12Rβ2 expression in CD8 T cells expressing the CD226 risk allele, suggesting an amplification in the loop STAT4-IL12Rβ2-IFNγ. Of interest, IL-12R and STAT4 are also known as genetic risk variants for MS, suggesting a coordinated involvement of this pathway in MS pathogenesis.^35^

Besides MS,^12^ the genetic variant of CD226 (rs763361T; 307Ser) is also associated with an increased risk of type 1 diabetes,^36^ rheumatoid arthritis,^37^ neuromyelitis optica,^38^ primary immune thrombocytopenia,^39^ juvenile idiopathic arthritis,^40^ Wegener granulomatosis,^41^ and autoimmune thyroid disease.^42^ Of note, as for MS, there is also strong evidence that CD8 T cells play a prominent role in some of these human diseases associated with the risk variant of CD226 such as type 1 diabetes and^43^ rheumatoid arthritis.^44^ Our study shows that CD8 T cells from donors carrying the CD226 risk variant produce high levels of IFNγ and this mechanism could be part of the immune dysregulation that contributes to the pathogenesis of these immune-mediated diseases. Thus, these findings provide important novel information about the potential mechanisms by which rs763361 affects CD226 signaling and CD8 T-cell functions.

## Appendix Authors

**Table:**

Name	Location	Contribution
Elena Morandi	Infinity-Toulouse Institute for Infectious and Inflammatory Diseases, Institut National de la Santé et de la Recherche Médicale (INSERM) UMR 1291, Centre National de la Recherche Scientifique (CNRS) UMR 5051, Université Paul Sabatier (UPS), Toulouse, France	Drafting/revision of the manuscript for content, including medical writing for content; major role in the acquisition of data; study concept or design; analysis or interpretation of data
Véronique Adoue, PhD	Infinity-Toulouse Institute for Infectious and Inflammatory Diseases, Institut National de la Santé et de la Recherche Médicale (INSERM) UMR 1291, Centre National de la Recherche Scientifique (CNRS) UMR 5051, Université Paul Sabatier (UPS), Toulouse, France	Analysis or interpretation of data
Isabelle Bernard, PhD	Infinity-Toulouse Institute for Infectious and Inflammatory Diseases, Institut National de la Santé et de la Recherche Médicale (INSERM) UMR 1291, Centre National de la Recherche Scientifique (CNRS) UMR 5051, Université Paul Sabatier (UPS), Toulouse, France	Study concept or design; analysis or interpretation of data
Ekaterina Friebel, PhD	Institute of Experimental Immunology, University of Zurich, Switzerland	Study concept or design; analysis or interpretation of data
Nicolas Nunez, PhD	Institute of Experimental Immunology, University of Zurich, Switzerland	Study concept or design; analysis or interpretation of data
Yann Aubert, PhD	Infinity-Toulouse Institute for Infectious and Inflammatory Diseases, Institut National de la Santé et de la Recherche Médicale (INSERM) UMR 1291, Centre National de la Recherche Scientifique (CNRS) UMR 5051, Université Paul Sabatier (UPS), Toulouse, France	Analysis or interpretation of data
Frederick Masson, PhD	Infinity-Toulouse Institute for Infectious and Inflammatory Diseases, Institut National de la Santé et de la Recherche Médicale (INSERM) UMR 1291, Centre National de la Recherche Scientifique (CNRS) UMR 5051, Université Paul Sabatier (UPS), Toulouse, France	Study concept or design
Anne S. Dejean, PhD	Infinity-Toulouse Institute for Infectious and Inflammatory Diseases, Institut National de la Santé et de la Recherche Médicale (INSERM) UMR 1291, Centre National de la Recherche Scientifique (CNRS) UMR 5051, Université Paul Sabatier (UPS), Toulouse, France	Study concept or design
Burkhard Becher, PhD	Institute of Experimental Immunology, University of Zurich, Switzerland	Study concept or design
Anne Astier, PhD	Infinity-Toulouse Institute for Infectious and Inflammatory Diseases, Institut National de la Santé et de la Recherche Médicale (INSERM) UMR 1291, Centre National de la Recherche Scientifique (CNRS) UMR 5051, Université Paul Sabatier (UPS), Toulouse, France	Study concept or design
Ludovic Martinet, PhD	Cancer Research Center of Toulouse (CRCT), Institut National de la Santé et de la Recherche Médicale (INSERM) UMR 1037, Centre National de la Recherche Scientifique (CNRS), Université Paul Sabatier (UPS), Toulouse, France	Study concept or design; analysis or interpretation of data
Abdelhadi Saoudi, PhD	Infinity-Toulouse Institute for Infectious and Inflammatory Diseases, Institut National de la Santé et de la Recherche Médicale (INSERM) UMR 1291, Centre National de la Recherche Scientifique (CNRS) UMR 5051, Université Paul Sabatier (UPS), Toulouse, France	Drafting/revision of the manuscript for content, including medical writing for content; study concept or design; analysis or interpretation of data

## Glossary

AKT: serine/threonine protein kinase
CM: central memory
EM: effector memory
ERK: extracellular signal-regulated kinase
FLT1: Fms-related receptor tyrosine kinase 1
MAPK: mitogen-activated protein kinase
MFI: mean fluorescent intensity
MS: multiple sclerosis
OSM: oncostatin M
PBMC: peripheral blood mononuclear cells
SNP: single-nucleotide polymorphism
SPHK1: sphingosine kinase 1
TCR: T-cell receptor
